# Book Reviews

**Published:** 1917-05

**Authors:** 


### BOOK REVIEWS

### INTERNATIONAL CLINICS.

A quarterly of illustrated clinical lectures and especially prepared original articles on treatment, medicine, neurology, paediatrics, obstetrics, gynecology, orthopaedics, pathology, dermatology, opthalmology, otology, rhinology, laryngology, hygiene, and other topics of interest to students and practitioners by leading members of the medical profession throughout the world edited by H. R. M. Landis, M.D., Philadelphia, U. S. A., with the collaboration of Chas. H. Mayo, M.D., Rochester, Sir Wm. Osler, Bart, M.D., F. R. S. Oxford, Rupert Blue, M.D., D. P. H., Washington, D. C., Frank Billings, M.D. Chicago, John C. Clark, M.D., Philadelphia, A. McPhedran, M. D., Toronto, James J. Walsh, M.D. New York, J. W. Ballantyne, M.D., Edinburgh, Charles Greene Cumston, M.D. Geneva, Arthur F. Biefeld, M.D., Chicago; Richard Kretx, M.D., Vienna; with correspondents in Montreal, London, Paris, Berlin, Vienna, Leipsic, Brussels, and Geneva. Volume II. Twenty-seventh series, 1917. Philadelphia and London, J. B. Lippincott Co.

This well-known quarterly contains much valuable and practical material by many well-known physicians and surgeons.

---

DISEASES OF THE GENITO-URINARY ORGANS AND THE KIDNEYS. By Robert H. Green, M.D., Professor of Genito-Urinary Surgery at the Fordham University, New York; and Harlow Brooks, M.D., Professor of Clinical Medicine, University and Bellevue Hospital Medical College. Fourth Edition thoroughly revised. Octavo of 666 pages, 301 illustrations. Philadelphia and London, W. B.

Saunders Company, 1917. Cloth, $5.50 net; Half Morocco, $7.00 net.

Greene and Brooks have written a very useful book which presents the convention standardized views of uro-genital surgery. The small amount of space devoted to sexual diseases makes the book of more value to the surgeon than to the general practitioner, otherwise the book, being a conjoint product of a surgeon and a physician, is a very valuable and useful one.

